# Peptidoglycan Recognition Proteins (PGRPs) Modulates Mosquito Resistance to Fungal Entomopathogens in a Fungal-Strain Specific Manner

**DOI:** 10.3389/fcimb.2019.00465

**Published:** 2020-01-23

**Authors:** José L. Ramirez, Ephantus J. Muturi, Lina B. Flor-Weiler, Karl Vermillion, Alejandro P. Rooney

**Affiliations:** ^1^Crop Bioprotection Research Unit, United States Department of Agriculture, National Center for Agricultural Utilization Research, Agricultural Research Service, Peoria, IL, United States; ^2^Functional Foods Research Unit, United States Department of Agriculture, National Center for Agricultural Utilization Research, Agricultural Research Service, Peoria, IL, United States

**Keywords:** antifungal immunity, PGRP, entomopathogenic fungi, mosquito immunity, *Aedes aegypti*

## Abstract

Fungal entomopathogens are potential tools for the control of mosquito vectors that transmit infectious agents that cause disease in humans and animals. During the infection process, effective recognition of the invading fungi by the mosquito, is a crucial step in mounting an appropriate anti-fungal response. In this study, we investigated the role of peptidoglycan recognition receptors (PGRPs) in host resistance to fungal entomopathogens at the early stages of infection. Our study identified the induction of *PGRP-LA, -LB, -LD, -LE*, and *-S1* during infection with two different fungal entomopathogenic strains. Furthermore, our data shows temporal differences in PGRP elicitation, with most PGRPs displaying significant upregulation at 60 h post-infection. Depletion of certain PGRPs via RNAi silencing resulted in a significant increase in fungal proliferation and a reduction in mosquito survival that was fungal strain-specific. Our data indicates that PGRPs play an important role in the antifungal response and expands our understanding of the factors that determine host susceptibility to fungal entomopathogens.

## Introduction

The yellow fever mosquito, *Aedes aegypti*, is one of the most important arboviral vectors, responsible for transmitting dengue, Zika and yellow fever virus (Chouin-Carneiro and Santos, 2017; Barzon, 2018; Braack et al., 2018). Fungal-based biopesticides are currently being considered as alternative tools to synthetic insecticides for the control of mosquito populations (Alkhaibari et al., 2016; Deng et al., 2019; Lovett et al., 2019). During the fungal infection process, a complex network of molecular interactions occurring between the invading fungi and the arthropod host determines the outcome of infection (Butt et al., 2016; Ramirez et al., 2018a). In this regard, pathogen recognition is one of the most critical aspects of immune activation and essential for a proper host defense (Wang et al., 2015; Lu and St Leger, 2016).

Although solely relying on an innate immune system, insects have evolved to effectively recognize foreign organisms (Schmidt et al., 2008). The detection of invading microbes is conducted by a series of specialized proteins known as pattern recognition receptors (PRR) that bind to pathogen-associated molecular patterns (PAMPs). This, in turn, allows them to distinguish pathogenic from non-pathogenic microbes, thus maintaining homeostasis (Bosco-Drayon et al., 2012; Hillyer, 2016). Mosquito PRRs include gram-negative binding proteins, fibrinogen-related proteins, the thioester-containing proteins, C-type lectins, leucine-rich repeat containing proteins, immunoglobulin domain proteins, Nimrod proteins, down syndrome cell adhesion molecules, and peptidoglycan recognition proteins (PGRPs) (Hillyer, 2016).

PGRPs are a class of proteins present across insects, mollusks and mammals. Although originally identified and named for their ability to detect peptidoglycan (a component of bacterial cell walls), recent research has found that these recognition proteins play important roles against other invading microbes such as protozoan parasites (Meister, 2006; Gendrin et al., 2017; Song et al., 2018). PGRPs can be divided based on their function (catalytic or non-catalytic) and on their transcript length (short PGRPs, PGRP-S, and long PGRPs, PGRP-L) (Dziarski and Gupta, 2018). Catalytic PGRPs have the ability to hydrolyze peptidoglycan into non-immunogenic molecules, thus preventing non-pathogenic organisms from over-activating the immune system. On the other hand, non-catalytic PGRPs bind to PAMPs and activate immune responses via the Toll and Imd immune pathways (Dziarski and Gupta, 2018). The *Aedes aegypti* mosquito genome has seven PGRP genes (Wang et al., 2018). Among them, *PGRP-LA, -LC, -LD* are non-catalytic with predicted transmembrane domains; while *PGRP-LB* and *PGRP-SC2* are catalytic and predicted to be secreted (Wang and Beerntsen, 2015; Wang et al., 2018).

Studies conducted in *Drosophila* and mosquitoes have shown that *PGRP-LC* plays a critical role in bacterial clearance and anti-*Plasmodium* defense (Meister, 2006; Meister et al., 2009). In turn, *PGRP-LA* has been found to positively regulate immunity against *Plasmodium* via activation of the Imd pathway (Gendrin et al., 2017). Although not much is known about *PGRP-LD*, bioassays conducted with the mosquito *Armigedes subaltus* indicated that it positively regulates the production of antimicrobial peptides (AMPs) (Wang and Beerntsen, 2015). The two catalytic PGRPs (*PGRP-LB* and *PGRP-SC2*), are thought to function as negative regulators of immune responses given that its knock-down increased the production of several AMPs (Wang and Beerntsen, 2015; Wang et al., 2018). *PGRP-LE* is known as an intracellular recognition molecule and has been found to modulate immune responses that facilitate *Wolbachia* colonization in *Ae. aegypti* mosquitoes (Pan et al., 2017). Its *Drosophila* ortholog has also been found to extracellularly activate the Imd pathway and the pro-phenoloxidase cascade (Kurata, 2014).

In this study we explored the role of PGRPs in modulating the mosquito antifungal response by evaluating gene expression and through RNAi-guided depletion of PGRPs, under the context of a fungal infection. Our study shows that PGRPs are key players in the mosquito resistance to fungal infection, with their effectiveness varying according to the invading fungal strain. This study provides a new perspective on the role of PGRPs in fungal detection and host response and expands our understanding of host-pathogen interactions, particularly anti-fungal immunity, with implications for our understanding of mosquito susceptibility to fungal entomopathogens.

## Materials and Methods

### Mosquito Rearing

*Aedes aegypti* (Rockefeller strain) colony was reared at 27°C and 80% humidity with a 12 h:12 h light/dark cycle. The colony was maintained by feeding adult females via an artificial feeding system using citrated bovine blood (HemoStat Laboratories Inc.). Adults were provided with a 10% sucrose solution and larvae were reared on a mixture of rabbit food and tropical fish food. Mosquitoes used in all experimental assays were conducted using 3- to 5-day old adult females.

### Fungal Strains and Infection Assays

Fungal infection assays were conducted as previously described (Ramirez et al., 2019). Briefly, fungal cultures of *Beauveria bassiana* (MBC 076) and *Isaria javanica* (ARSEF 5874) were grown on ¼ strength Sabouraud dextrose agar and yeast extract (SDAY) medium at 26°C for 15-days. Spore oil formulations were prepared as previously reported (Ramirez et al., 2018a). Briefly, fungal spores were harvested from 15-day old cultures with soy bean oil as a carrier. The mixture was briefly homogenized with an electronic pestle and then filtered through cheese cloth. An improved Neubauer hemocytometer was used to count and adjust conidial concentrations to 1 × 10^8^ conidia/mL. Topical infection assays were conducted by applying 50.6 nL of the conidial suspension on the central surface of the coxal region of cold-anesthetized mosquitoes using a Nanoject II micropipet. This corresponded to an estimated 50,600 conidia per mosquito. The control group was exposed to the same volume of soy bean oil without fungal spores. Three independent cohorts per treatment were used and the experiment was replicated in at least two independent experiments. New batches of mosquitoes and fresh conidial suspensions were used for each experiment. All treated mosquitoes were transferred to an insect cup-cage and maintained under standard insectary conditions and provided with 10% sucrose solution until the end of the experiment. Mortality was monitored daily and all mosquito cadavers were removed from the cages. Mosquito survival curves were analyzed using the Kaplan-Meier estimator with median survival time differences between each treatment compared via Log-rank test (GraphPad Prism 8.0). The LT50 and LT95 values were calculated by probit analysis using SAS 9.4 statistical package.

### Evaluation of Gene Expression

For gene expression analyses, pools of five challenged mosquitoes were collected at 48, 60, and 72 h post-infection (PI). These time points were selected to assess the progression of early immune elicitation when infected by these two different entomopathogenic fungal species. RNA was extracted from samples using TRIzol (Invitrogen) according to the manufacturer's instructions. Concentration and quality of the extracted RNA were evaluated via NanoDrop (Thermo Scientific). Synthesis of cDNA was conducted on normalized amounts of RNA using the QuantiTec reverse transcription kit with DNA Wipeout (Qiagen). Quantitative real-time PCR to amplify seven PGRPs (*PGRP-LA, PGRP-LB, PGRP-LC, PGRP-LD, PGRP-LE, PGRP-S1, and PGRP-SC2*), six GNBPs (*GNPS-1* through *GNBP-6*) and two molecules known to be elicited in response to fungal infection (*CLSP2* and *TEP22)* was conducted in a 10 μl reaction using one microliter of the generated cDNA. The PowerUp SYBR green Master mix qPCR kit (Qiagen) was used in all reactions with gene specific primers (Table S1). RT-qPCR cycling conditions were those recommended by the manufacturer and consisted of holding at 95.0°C for 10 min and 40 cycles of 15 s at 95.0°C and 1 min at 60°C. Melt curve analysis was included at the end of each qPCR run. Gene expression was evaluated in at least four pools per treatment group, with each sample analyzed in duplicate (technical replicates) and the reproducibility of the results evaluated in at least two independent experiments, each conducted with new batches of mosquitoes and fresh conidial suspensions. The expression level of PGRPs and other target genes were normalized using the ribosomal protein Rps17 (AAEL004175) as a reference gene (Dzaki et al., 2017; Ramirez et al., 2018b). *Rps17* has been routinely used in expression profiles involving *Aedes aegypti* (Barletta et al., 2012; Ramirez et al., 2018b). The real-time qPCR reaction was conducted on an Applied Biosystems QuantStudio 6 Flex Real-time PCR system (ThermoFisher Scientific). Gene expression profiles were evaluated post run using the ΔΔCt method (Livak and Schmittgen, 2001). The statistical significance of relative expression was determined on log_2_-transformed ΔΔCt values via ANOVA with Dunnett's post-test in Prism 8 (GraphPad).

### Gene Silencing

The transient depletion of PGRP genes was conducted via RNA interference (RNAi)-directed silencing as previously described (Ramirez et al., 2012). In short, the T7 promoter was added to each target gene primer to generate amplicons around 400-bp using cDNA templates from *Ae. aegypti*. The HiScribe T7 Quick High Yield RNA synthesis kit (New England Biolabs) was used to generate dsRNA products from each target gene and each product was then diluted with molecular-grade water to a 3 μg/μl solution. Cold anesthetized 2- to 3-day old *Ae. aegypti* females were injected with 69 nl of a 3 μg/μl dsRNA solution into the thorax using a nano-injector (Nanojet III, Drummond Scientific). For infection assays evaluating the effect of silencing on mosquito survival, mosquitoes were challenged with entomopathogenic fungi at 1-day post-dsRNA injection. Silencing efficiency relative to *dsFluc* controls was evaluated for each silenced gene in mosquito whole-bodies at 3–4-days post-injection. We conducted a triple silencing of the three most highly expressed PGRPs (*PGRP-LA, PGRP-LD*, and *PGRP-S1*), to overcome a potential redundancy in PGRP-derived protection. For the triple-knockdown, dsRNA products for each target gene were combined to a final concentration of 3 μg/μl each and with a final concentration of 9 μg/μl dsRNA injected per mosquito. To account for the total dsRNA product injected into ds*PGRP*-treated mosquitoes, control mosquitoes were also injected with 69 nl of a 9 μg/μl of ds*Fluc* solution. Primers designed for gene silencing are reported in Table S1.

### Statistical Analyses

Graphs and statistical analysis for survival curves and gene expression were conducted using the software Prism 8 (GraphPad). Significance of survival curves was assessed using the Kaplan-Meier estimator with Log-rank test, while gene expression values were compared via ANOVA with Dunnett's post-test. LT_50_ and LT_95_ were evaluated via probit analysis using SAS 9.4 statistical package. Statistical significance was assessed at *P* < 0.05, with the strength of the significance represented with asterisks (^*^P < 0.05; ^**^P < 0.01; ^***^P < 0.001). The error bars indicate the standard error of the mean and the type of test used is described in the respective figure legend.

## Results

### *Ae. aegypti* PGRPs Are Differentially Elicited by Fungal Entomopathogenic Infection in a Time and Strain-Specific Manner

**Figure 1:**
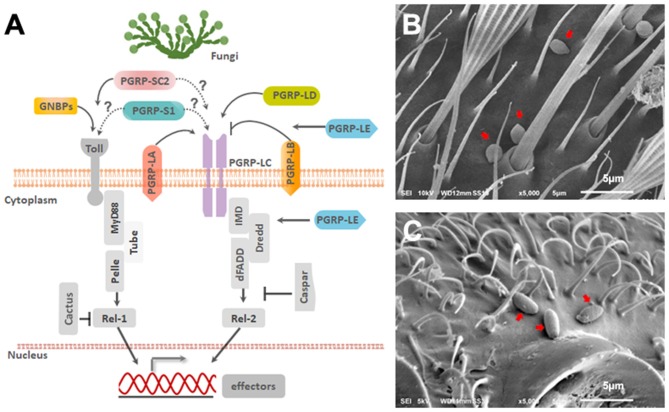
*Ae. aegypti* PGRPs in relation to immune signaling pathways. **(A)** Schematic diagram depicting the predicted location of PGRPs and their interaction with downstream molecules based on studies from mosquitoes and *Drosophila*. Scanning Electron Microscopy imaging of **(B)** *B. bassiana* and **(C)** *I. javanica* conidia on the mosquito cuticle at 24 h PI. White line on the bottom of the image is 5 μm.

**Figure 2:**
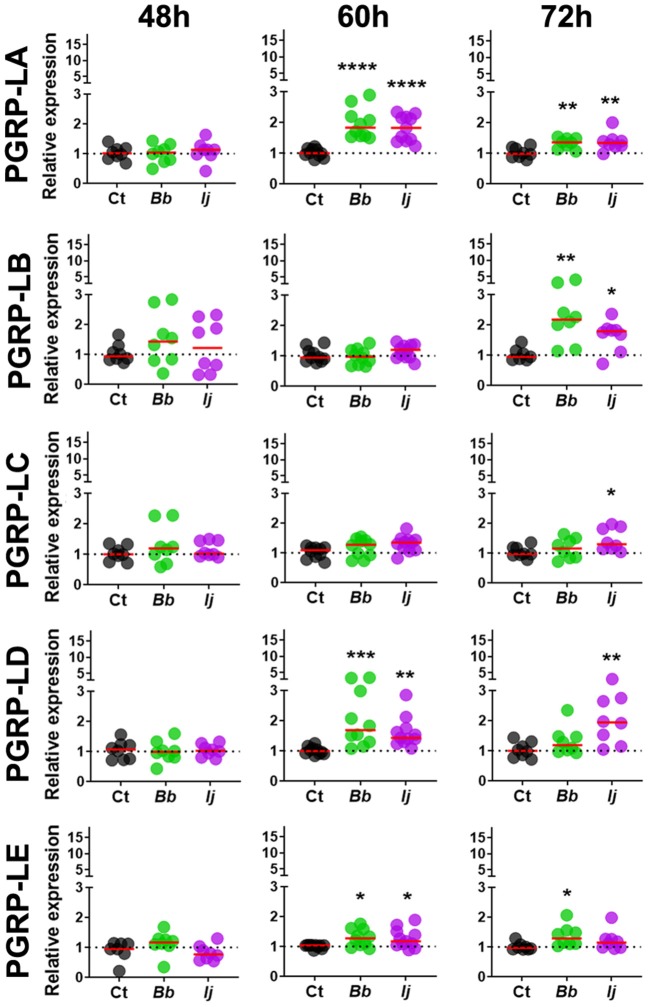
Induction of long PGRPs in *Ae. aegypti* mosquitoes at the early stages of fungal infection. Gene expression profiles of *PGRP-LA, PGRP-LB, PGRP-LC, PGRP-LD*, and *PGRP-LE* in mosquito whole-bodies infected with either *B. bassiana* or *I. javanica*, evaluated at 48, 60, and 72 h PI. Each dot represents a pool of five mosquitoes and the horizontal red bar indicates the median level of expression from two independent experiments. The statistical significance of fold change values was determined on log_2_-transformed values via ANOVA followed by Dunn's multiple comparison test. *P < 0.05, **P < 0.01, ***P < 0.001, ****P < 0.0001.

To evaluate whether *Ae. aegypti* PGRPs can respond to entomopathogenic fungal infection, mosquitoes were challenged with either soybean oil, *B. bassiana* or *I. javanica*. Samples were collected at 48, 60, and 72 h post-infection (PI) and the expression of seven PGRPs (*PGRP-LA, PGRP-LB, PGRP-LC, PGRP-LD, PGRP-LE, PGRP-S1*, and *PGRP-SC2*) (Figure 1) were evaluated via qPCR. Our expression analyses showed that *PGRP-LA* elicitation was absent at 48 h PI but strongly upregulated at 60 and 72 h PI. This pattern of gene induction was similar in mosquitoes infected with either *B. bassiana* or *I. javanica* (Figure 2). In contrast, *PGRP-LB* was only elicited at the latest time point, 72 h PI, in both *B. bassiana* and *I. javanica*-infected mosquitoes.

**Figure 3:**
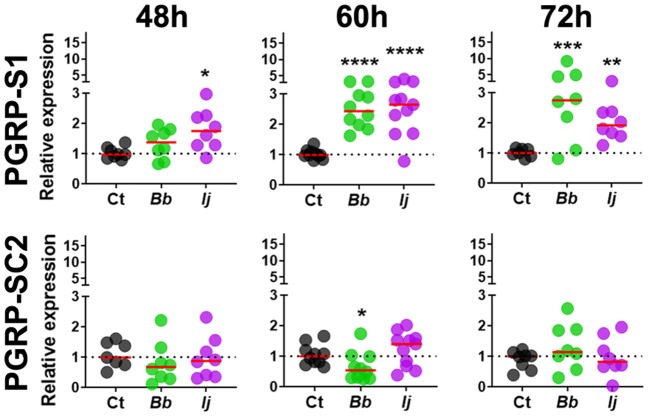
Induction of short PGRPs in *Ae. aegypti* mosquitoes at the early stages of fungal infection. Gene expression profiles of *PGRP-S1* and *PGRP-SC2* in mosquito whole-bodies infected with either *B. bassiana* or *I. javanica*, evaluated at 48, 60, and 72 h PI. Each dot represents a pool of five mosquitoes and the horizontal red bar indicates the median level of expression from two independent experiments. The statistical significance of fold change values was determined on log_2_-transformed values via ANOVA followed by Dunn's multiple comparison test. *P < 0.05, **P < 0.01, ***P < 0.001, ****P < 0.0001.

Gene expression analysis of *PGRP-LC* indicated absence of elicitation at 48 and 60 h PI but displayed significant increase in expression at 72 h PI only in mosquitoes infected with *I. javanica* (Figure 2). We observed a more sustained expression *PGRP-LD*, in a pattern similar to that of *PGRP-LA*, with absent elicitation at 48 h but with strong induction at 60 h in mosquitoes infected with either fungal entomopathogenic strain. This *PGRP-LD* expression changed by 72 h PI, when mosquitoes infected with *I. javanica* maintained *PGRP-LD* induction while those infected with *B. bassiana* displayed a drop in significant gene expression from the one presented at 60 h PI. *PGRP-LE* expression was absent at 48 h, but we observed a slight but significant upregulation in both infected groups at 60 h PI (Figure 2). This expression pattern changed by 72 h PI when only *B. bassiana*-infected mosquitoes presented significant *PGRP-LE* elicitation. We also evaluated the expression patterns of the two short-PGRPs (*PGRP-S1* and *PGRP-SC2*) during the early time of infection. Our data shows that *PGRP-S1* was the only PGRP that displayed consistently higher levels of gene expression throughout the time points of infection (Figure 3). This induction started at 60 h PI and continued at 72 h PI for *B. bassiana*-infected mosquitoes. Meanwhile, *PGRP-S1* induction for *I. javanica*-infected mosquitoes started at least 12 h earlier, at 48 h PI and was sustained through 60 and 72 h PI. In comparison, *PGRP-SC2* was the only PGRP that did not show any significant upregulation during the time points evaluated (Figure 3). Instead, *PGRP-SC2* displayed a slight but significant down regulation at 60 h PI but only in *B. bassiana-infected* mosquitoes.

### GNBPs Are Differentially Regulated Throughout the Early Time Points of Infection

**Figure 4:**
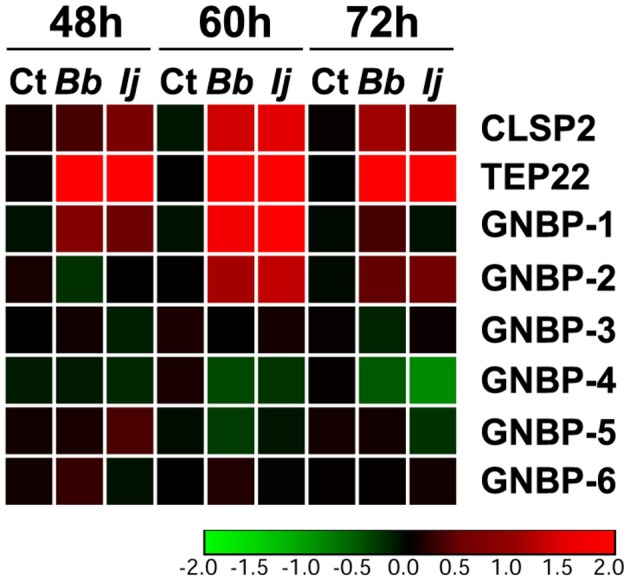
Temporal elicitation of GNBPs and other fungal recognition molecules. Heatmap generated from the gene expression patterns of GNBPs, *TEP22*, and *CLSP2* at 48, 60, and 72 h PI. Heatmap represents the median log_2_ fold change values from two independent experiments. The color red represents upregulation and green downregulation in comparison to the controls.

We next evaluated the expression profile of GNBPs to compare to that of PGRPs, since a few of their members were found to recognize fungal infections (Matskevich et al., 2010). Our analysis included *GNBP-1* through *GNBP-6*, with gene expression evaluated at the same time points specified for PGRPs. Our gene expression analyses indicated a significant upregulation of only two *GNBP* genes. For instance, we observed a significant *GNBP-1* elicitation at 48 and 60 h PI for mosquitoes infected with either fungal entomopathogen. However, this increase in expression subsided by 72 h PI in all challenged groups. *GNBP-2* followed a similar pattern, with absent induction at 48 h but with a robust significant increase in gene expression at 60 and 72 h PI for mosquitoes infected with either entomopathogenic fungi. In contrast, we observed a significant downregulation in the expression of *GNBP-4* in *I. javanica*-infected mosquitoes at 72 h PI (Figure 4, Figures S1, S2). No other GNBP gene displayed significant regulation during the time points tested.

To compare the levels of expression of these important pathogen recognition receptors, we also measured two molecules that have been previously found to be elicited in response to fungal infection, *CLSP2* and *TEP22* (Wang et al., 2015; Ramirez et al., 2018a). Our results indicate a significant elicitation of *CLSP2* at 48 h PI in *I. javanica*-infected mosquitoes and at 60 and 72 h in mosquitoes infected with either fungi. In comparison, *TEP22* displayed a robust significant elicitation across all three time points and in mosquitoes infected with either fungal strain (Figure 4, Figure S1).

### PGRP Silencing Affects Mosquito Survival to Fungal Infection in a Fungal Species-Specific Manner

To further evaluate the role of PGRP in the antifungal defense, we conducted a RNAi-based knockdown of six different PGRPs prior to infection with entomopathogenic fungi. Gene expression of each PGRP target, at 3-days post-silencing, indicated a reduction of 80% for ds RNA *PGRP-LA*, 40% for dsRNA *PGRP-LC*, 33% for dsRNA *PGRP-LD*, 46% for dsRNA *PGRP-LE*, 83% for dsRNA *PGRP-S1*, and 38% for dsRNA *PGRP-SC2*. We were unable to successfully silence *PGRP-LB* and was not included in further analysis. Gene expression of each PGRP was also evaluated in each single PGRP knockdown to evaluate potential induction due to functional redundancy. This assessment indicated significant induction of *PGRP-LD* in mosquitoes where *PGRP-LA* and *PGRP-LC* were silenced (Figure S3). In addition, *PGRP-SC2* was significantly induced in mosquitoes with *PGRP-LC* knockdown. No other PGRP induction was observed with any of the remaining knockdown PGRP targets (Figure S3).

**Figure 5:**
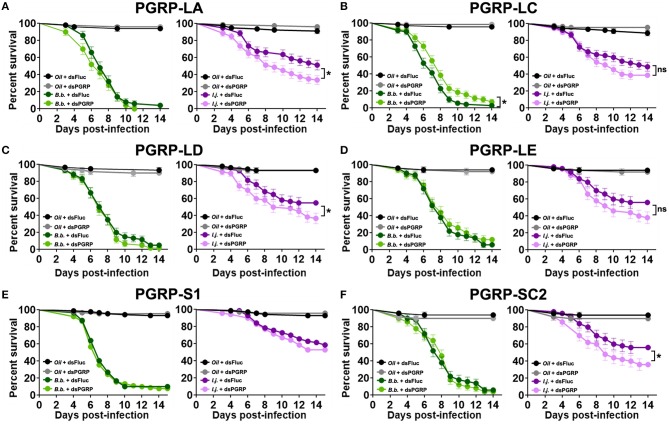
PGRP depletion has a detrimental effect on the survival of mosquitoes infected with the fungal entomopathogen *I. javanica*. Survival curves of dsRNA-treated mosquitoes with knockdown of **(A)** *PGRP-LA*, **(B)** *PGRP-LC*, **(C)** *PGRP-LD*, **(D)** *PGRP-LE*, **(E)** *PGRP-S1*, and **(F)** *PGRP-SC2* following infection with either *B. bassiana* or *I. javanica*. Survival curves represents at least four independent experiments and data was analyzed with Log-rank Test (GraphPad Prism 8). Error bars indicate the SEM. *P < 0.05.

Mosquito survival following fungal challenge differed with each silenced PGRP and also with each fungal entomopathogenic species. We did not observe a significant effect on the survival of *B. bassiana*-infected mosquitoes in which *PGRP-LA* was depleted (Log-rank Mantel-Cox test, *X*^2^ = 1.59, *P* = 0.21) (Figure 5A). Interestingly, depletion of *PGRP-LC* led to a slight but significant increase in survival compared to the controls when challenged with *B. bassiana* (Log-rank Mantel-Cox test, *X*^2^ = 5.88, *P* = 0.015). Silencing of the remaining PGRPs displayed similar phenotypes to that of ds*PGRP-LA*, with no significant effect on the survival of *B. bassiana*-infected mosquitoes in which *PGRP-LD* (Log-rank Mantel-Cox test, *X*^2^ = 0.74, *P* = 0.39) (Figure 5C)*, PGRP-LE* (Log-rank Mantel-Cox test, *X*^2^ = 0.58, *P* = 0.45) (Figure 5D), *PGRP-S1* (Log-rank Mantel-Cox test, *X*^2^ = 0.47, *P* = 0.49) (Figure 5E), or *PGRP-SC2* (Log-rank Mantel-Cox test, *X*^2 =^ 0.03, *P* = 0.86) (Figure 5F), had been depleted.

In comparison, *I. javanica*-challenged mosquitoes showed a significant reduction in survival compared to ds*Fluc* controls, with the depletion of either *PGRP-LA* (Log-rank Mantel-Cox test, *X*^2^ = 5.85, *P* = 0.015) (Figure 5A), *PGRP-LD* (Log-rank Mantel-Cox test, *X*^2^ = 4.43, *P* = 0.035) (Figure 5C), or *PGRP-SC2* (Log-rank Mantel-Cox test, *X*^2^ = 4.14, *P* = 0.042) (Figure 5F). No significant change in survival was observed with the depletion of either *PGRP-LC* (Log-rank Mantel-Cox test, *X*^2^ = 1.67, *P* = 0.197) (Figure 5B), *PGRP-LE* (Log-rank Mantel-Cox test, *X*^2^ = 2.93, *P* = 0.087) (Figure 5D), or *PGRP-S1* (Log-rank Mantel-Cox test, *X*^2^ = 2.50, *P* = 0.114) (Figure 5E, Table 1).

**Table 1:** Estimated LT_50_ and LT_95_ values from *B. bassiana and I. javanica-*infected mosquitoes in which *Fluc* (control) or a given *PGRP* transcript was depleted via RNAi.

	**Fungal strain**			
	***B. bassiana*** **(MBC 076)**		***I. javanica*** **(ARSEF 5875)**	
**RNAi target**	**LT_**50**_ (95% CI)**	**LT_**95**_ (95% CI)**	**LT_**50**_ (95% CI)**	**LT_**95**_ (95% CI)**
*Fluc*	7.5 (6.9–7.6)	11.1 (10.6–11.7)	12.6 (11.9–13.5)	ND
*PGRP-LA*	6.4 (6.1–6.7)	9.9 (9.4–10.5)	9.7 (9.3–10.3)	ND
*Fluc*	6.6 (6.3–6.9)	11.0 (10.5–11.5)	12.1 (11.4–12.8)	ND
*PGRP-LC*	7.8 (7.5–8.1)	13.0 (12.4–13.7)	10.3 (9.9–10.8)	ND
*Fluc*	7.4 (7.1–7.8)	12.4 (11.8–13.1)	12.5 (11.8–13.4)	ND
*PGRP-LD*	7.1 (6.8–7.0)	11.5 (11.0–12.1)	10.4 (9.8–11.1)	ND
*Fluc*	7.7 (7.4–8.1)	12.7 (12.1–13.5)	13.7 (12.8–14.9)	ND
*PGRP-LE*	8.2 (7.8–8.6)	13.9 (12.1–14.8)	10.7 (10.1–11.4)	ND
*Fluc*	7.7 (7.4–8.0)	12.8 (12.3–13.3)	15.5 (14.3–17.2)	ND
*PGRP-S1*	7.3 (7.0–7.5)	12.1 (11.7–12.7)	13.0 (12.2–14.1)	ND
*Fluc*	7.8 (7.4–8.1)	12.7 (12.1–13.5)	13.7 (12.8–14.9)	
*PGRP-SC2*	7.4 (7.1–7.8)	12.2(11.6–13.0)	10.1 (9.5–10.8)	ND
*Fluc*	7.5 (7.2–7.8)	13.2 (12.6–13.9)	11.3 (10.7–12.0)	ND
*3xPGRP*	5.8 (5.6–6.0)	9.1 (8.7–9.6)	8.5 (8.0–9.0)	ND

*ND (Not determined) denotes treatments where the LT values could not be estimated, due to low mortality*.

**Figure 6:**
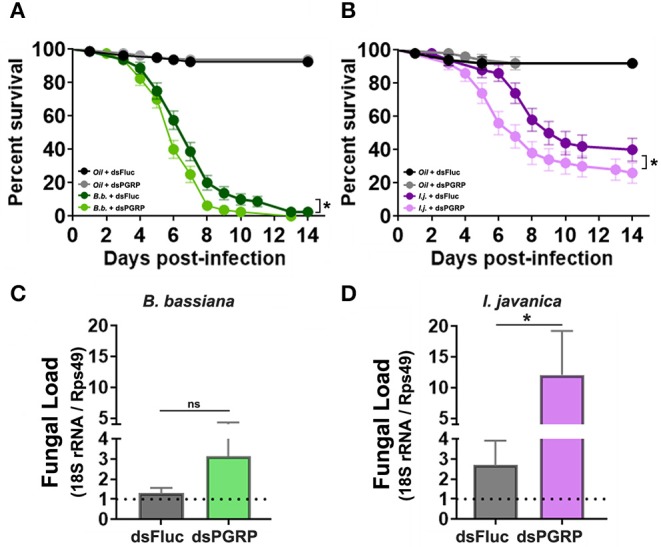
Triple-knockdown of PGRPs leads to a decrease in mosquito survival and increases fungal proliferation. Survival curves of triple-silenced mosquitoes (targeting *PGRP-LA, PGRP-LD*, and *PGRP-S1*) under the context of infection with either **(A)** *B. bassiana* or **(B)** *I. javanica*. Estimation of fungal loads in PGRP triple-silenced mosquitoes infected with **(C)** *B. bassiana* or **(D)** *I. javanica* at five days post-infection. Survival curves represents at least four independent experiments and data was analyzed with Log-rank Test (GraphPad Prism 8). Fungal loads were analyzed from two independent experiments. The statistical significance of fold change values was determined on log_2_-transformed values via Mann-Whitney test. Error bars indicate the SEM. *P < 0.05.

To circumvent any potential redundancy in PGRP-derived protection, we conducted a triple silencing of the three most highly elicited PGRPs (*PGRP-LA, PGRP-LD* and *PGRP-S1*) and assessed its effect on mosquito survival. The silencing efficiency observed in tripled-silenced mosquitoes was the same as the single-silenced targets (42% silencing of *PGRP-LD*, 50% silencing of *PGRP-LA* and 85% silencing of *PGRP-S1*). Triple silencing prior to fungal infection led to a slim, albeit significant decrease in mosquito survival compared to the control groups in mosquitos infected with *B. bassiana* (Log-rank Mantel-Cox test, *X*^2^ = 6.55, *P* = 0.011) (Figure 6A, Table 1). A much clearer and stronger phenotype was observed in *I. javanica*-infected mosquitoes (Log-rank Mantel-Cox test, *X*^2^ = 4.40, *P* = 0.036) (Figure 6B, Table 1).

To further assess whether PGRP knockdown increases fungal proliferation, we evaluated the levels of fungal loads via RT-qPCR in both fungal infections at 5-days post-infection. The evaluation of fungal loads in triple-silenced mosquitoes, followed similar trends to that of survival curves, with no major change in fungal loads in *B. bassiana*-infected mosquitoes (Figure 6C) compared to ds*Fluc* controls, but with a significant increase in *I. javanica*-infected mosquitoes (Figure 6D).

## Discussion

Successful mosquito immune protection against microbial pathogens relies on pathogen recognition, effective immune signaling and on anti-pathogenic effectors. In this regard, peptidoglycan recognition proteins (PGRPs) are a family of proteins whose diverse functions, covering these three areas, are used to maintain the homeostasis between resistance and tolerance to microorganisms (Dziarski and Gupta, 2006, 2018; Paredes Juan et al., 2011; Royet et al., 2011). In this study, we evaluated the participation of the PGRP family in antifungal immunity at the early stages of infection in the mosquito *Ae. aegypti*.

The first objective of our study was to assess the induction of PGRPs in mosquitoes topically-infected with two different species of entomopathogenic fungi, *B. bassiana* and *I. javanica*. The gene expression profile displayed temporal elicitation of six out of seven PGRPs assessed. Notably, our analyses indicate that, except for *PGRPS1*, most PGRPs are not elicited until around 60 h post-fungal infection. This could mean that either, their induction is dependent on fungal load or that further processing of fungal-derived PAMPs are needed upstream of PGRPs. This study did not evaluate these two possibilities and further studies are needed to unravel the mechanism behind PGRP induction upon fungal infection.

In comparison, similar gene expression patterns were observed with gram-negative binding proteins (GNBPs), another family of proteins associated with fungal recognition. Here, our results indicate an earlier triggering of *GNBP-1* (at 48 h PI) and a later induction of *GNBP-2* (at 60 h PI). These two GNBPs have been associated with the response to fungal infection (Aguilar et al., 2005; Wang et al., 2015). In addition, the *Drosophila GNBP-3*, an ortholog of *Ae. aegypti GNBP-2*, has been associated with fungal recognition (Gottar et al., 2006; Matskevich et al., 2010; Arvanitis et al., 2013). Our data also indicated the induction of another marker of fungal infection, *TEP22* (Wang et al., 2015), at 48 h PI. Further work is needed to determine whether GNBPs, TEPs and PGRPs share similar function in either fungal recognition or as anti-fungal effectors.

Our study revealed that some of these pathogen recognition receptors, such as *PGRP-LA, PGRP-LD*, and *PGRP-S1*, had a robust and significant increase in gene expression in most time points evaluated. This was observed independent of the strain of fungal entomopathogen infecting the mosquito. In general, most of these PGRPs have been associated with the Imd pathway, and its triggering is thought to occur via the detection of peptidoglycan (PGN) during bacterial infections (Zaidman-Rémy et al., 2006; Dziarski and Gupta, 2018). Fungal cells lack PGN in their cell walls, however, several studies have implicated PGRPs in the defense against other microbial organisms in a PGN-independent manner. For instance, *PGRP-LA2* from the mosquito *Anopheles gambiae* was predicted to not bind PGN but still had antiparasitic activity against the rodent malaria parasite *Plasmodium berghei* (Meister, 2006; Gendrin et al., 2017). In addition, subsequent studies in the malaria vector *An. coluzzii*, identified *PGRP-LA1* and *PGRP-S2/3* as critical in the defense against *Plasmodium* infection. Interestingly, a recent study found that *Ae. aegypti* mosquitoes challenged with gram positive and gram negative bacteria did not show the induction of *PGRP-LA* or *PGRP-LD* (Wang and Beerntsen, 2015). Thus, their significant elicitation in our study, following challenge with fungal entomopathogens, further suggests their role in recognizing fungal-derived molecules.

The PGRP ability to detect multiple pathogen-derived molecules might be inherent to their function and structural composition. Indeed, structural analysis of PGRP domains in *An. gambiae* and *Drosophila* points to different regions in the PGRP molecule with potential ability to distinguish other PAMPs such as 1,3-β-Glucan (Meister, 2006). For instance, a PGRP from the beetle *Holotrichia diomphalia* was found to recognize and bind 1,3-β-D-glucan and to induce proPO activation (Lee et al., 2004). The ability to detect polysaccharides such as β-glucans is essential for the proper recognition of fungi, given that most fungal cell walls are composed primarily of 1,3-β-Glucan and to a lesser extend chitin (Bowman and Free, 2006; Latgé, 2010; Snarr et al., 2017). Furthermore, our study revealed the elicitation of other known fungal-recognition molecules (such as *TEP22* and *CLSP2*) at the same time of infection, providing further support that the activation of mosquito PGRPs is triggered by fungal-derived molecules.

We next corroborated the role of PGRPs in the antifungal defense by depleting PGRP expression via RNAi prior to fungal infection. Our results suggest that the strength of the PGRP anti-fungal response varies according to the invading fungal strain, with PGRP-silencing significantly affecting survival in mosquitoes infected with *I. javanica* but with no effect in those infected with *B. bassiana*. This detrimental effect of PGRP depletion was observed in three out of six PGRPs tested. This included *PGRP-LA* and *PGRP-LD*, which displayed strong gene elicitation during the infection process.

An interesting phenotype observed in our assays was the detrimental effect of *PGRP-SC2* depletion despite the absence of a significant induction in gene expression. This phenotype could be due to its predicted function as a PGRP with amidase activity, acting as a negative regulator of the immune response. Thus, while constitutively expressed, at basal levels it might be involved in catalyzing fungal-derived molecules that could potentially lead to a deleterious overstimulation of the immune response. In fact, a previous study in *Drosophila*, demonstrated that removal of PGRPs with amidase activity led to the uncontrolled activation of the Imd pathway and reduced fly survival due to an excessive immune response (Paredes Juan et al., 2011). Further studies are needed to confirm this possibility in mosquitoes and to effectively characterize the function of this PGRP in pathogen detection and immune modulation.

The absence of any detrimental effect of PGRP silencing on survival of *B. bassiana*-infected mosquitoes is most likely reflective of the interaction of *B. bassiana* with PGRPs and its subsequent signaling through downstream immune signaling pathways. Hence, the observed phenotype depicts the relative importance of the mosquito immune pathways in the anti-*B. bassiana* defense repertoire. For instance, our prior study identified the Imd pathway as an important component of the mosquito antifungal response whose effectiveness was less pronounced in mosquitoes infected with *B. bassiana* (Ramirez et al., 2019). Given that most of these PGRPs, particularly *PGRP-LA* and *PGRP-LD*, have been linked to the Imd pathway, it is plausible that depletion of those pathogen recognition receptors will also show less pronounced effects on survival of *B. bassiana*-infected mosquitoes. Alternatively, it might also be due to the redundancy in *B. bassiana* detection by several PGRPs. Our triple-silenced assays targeting three of the most highly elicited PGRPs (*PGRP-LA, PGRP-LD*, and *PGRP-S1*) hint to this possibility, with a slight but significant reduction in survival in mosquitoes infected with *B. bassiana*. Furthermore, PGRP expression analysis in single PGRP knockdowns indicated the significant elicitation of *PGRP-LD* in mosquitoes in which *PGRP-LA* or *PGRP-LC* were silenced. This suggests partial redundancy in these PGRPs and agrees with what is known with respect to *PGRP-LA, PGRP-LD*, and *PGRP-LC*, as interacting partners of the Imd pathway.

The slight but significant increase in survival of *PGRP-LC*-depleted mosquitoes challenged with *B. bassiana* but absent in *I. javanica* infection is puzzling. It could indicate that elicitation of *PGRP-LC*, and thus Imd pathway activation, is somewhat counterproductive to mosquito survival under the context of a fungal infection. However, our previous study knocking down the Imd pathway transcription factor Rel2 depicted a reduction in survival, indicating a protective effect of Imd pathway activation (Ramirez et al., 2019). Thus, the observed phenotype could represent a specific interaction with *B. bassiana* at the level of *PGRP-LC* rather than a general mosquito antifungal response. The degree and specificity of this potential interaction between *B. bassiana* and *PGRP-LC* remains to be elucidated.

Further assays evaluating the fungal load in PGRP-depleted mosquitoes confirmed the participation of PGRPs in the antifungal host response, whose dynamics are in turn dependent on the strain of invading fungal entomopathogen. For instance, our study showed a much greater and significant increase in fungal load in mosquitoes infected with *I. javanica* than those infected with *B. bassiana*. This is in agreement with our survival phenotypes and reiterates that while fungal detection by PGRP, and its subsequent downstream signaling through the immune pathways, has a stronger protective effect on mosquitoes infected with *I. javanica*, it has a very limited effect on infection with *B. bassiana*. This likely suggests a stronger ability of *B. bassiana* to circumvent the mosquito antifungal immune response. Whether this is due to active immune-suppressive activity via secondary metabolites or due to the inherent resistance of *B. bassiana* to the immune-derived effectors produced via PGRP elicitation, remains to be elucidated. Furthermore, our study only evaluated two fungal entomopathogenic species and it is quite possible that different fungal strains of *B. bassiana* will present different infection phenotypes. Thus, further work evaluating the role of PGRPs in infections with different strains is needed to confirm whether this phenotypic response is solely a characteristic of *B. bassiana*.

In summary, our study reveals that in the complex interaction that exists between the mosquito immune system and the invading entomopathogenic fungi, PGRPs play a critical role in antifungal defense. Our data further indicates that the effectiveness of PGPR-based fungal detection/response varies according to the infecting fungal species and displays a temporal elicitation of PGRP genes during the early stages of infection. Information derived from our understanding of how mosquitoes detect and respond to microbes, and the mechanisms underlying host resistance to fungal entomopathogens, can improve mosquito control strategies using fungal entomopathogens by facilitating the selection of strains with different modes of action.

## Data Availability Statement

All datasets generated for this study are included in the article/Supplementary Material.

## Author Contributions

JR conceived and conducted the study, analyzed the data, and wrote the manuscript. EM assisted on the determination, analysis of time-dependent mortality data, and manuscript writing. LF-W and KV assisted with electron microscopy imaging and manuscript writing. AR assisted with data analysis and interpretation. All authors edited and approved the final manuscript.

### Conflict of Interest

The authors declare that the research was conducted in the absence of any commercial or financial relationships that could be construed as a potential conflict of interest.

## Supplementary Material

Figure S1 Gene expression profiles in mosquito whole-bodies infected with either *B. bassiana* or *I. javanica*, evaluated at 48, 60, and 72 h PI. Induction of **(A)** *CLSP2*, **(B)** *TEP22*, **(C)** *GNBP-1*, and **(D)** *GNBP-2* in *Ae. aegypti* mosquitoes at the early stages of fungal infection. Each dot represents a pool of five mosquitoes and the horizontal red bar indicates the median level of expression from two independent experiments. The statistical significance of fold change values was determined on log_2_-transformed values via ANOVA followed by Dunn's multiple comparison test. ^*^*P* < 0.05, ^**^*P* < 0.01, ^****^*P* < 0.0001.

Figure S2 Gene expression profiles in mosquito whole-bodies infected with either *B. bassiana* or *I. javanica*, evaluated at 48, 60, and 72 h PI. Induction of **(A)** *GNBP-3*, **(B)** *GNBP-4*, **(C)** *GNBP-5*, and **(D)** *GNBP-6* in *Ae. aegypti* mosquitoes at the early stages of fungal infection. Each dot represents a pool of five mosquitoes and the horizontal red bar indicates the median level of expression from two independent experiments. The statistical significance of fold change values was determined on log_2_-transformed values via ANOVA followed by Dunn's multiple comparison test. ^**^*P* < 0.01.

Figure S3 PGRP induction in mosquitoes with single knockdowns of Fluc (control), **(A)** *PGRP-LA*, **(B)** *PGRP-LC*, **(C)** *PGRP-LD*, **(D)** *PGRP-LE*, **(E)** *PGRP-S1*, or **(F)** *PGRP-SC2*. Gene expression profiles were conducted in mosquito whole-bodies at 3-days post dsRNA injection. The statistical significance of fold change values was determined on log_2_-transformed values via ANOVA followed by Dunn's multiple comparison test. ^*^*P* < 0.05, ^**^*P* < 0.01, ^***^*P* < 0.001.

Table S1 Primer sequences used in qPCR and RNAi assays.
