# Supplementary figures and images for: Peptidoglycan Recognition Proteins (PGRPs) Modulates Mosquito Resistance to Fungal Entomopathogens in a Fungal-Strain Specific Manner
Source: Front Cell Infect Microbiol. 2020 Jan 23;9:465. doi: 10.3389/fcimb.2019.00465 (PMC6989432; doi:10.3389/fcimb.2019.00465)

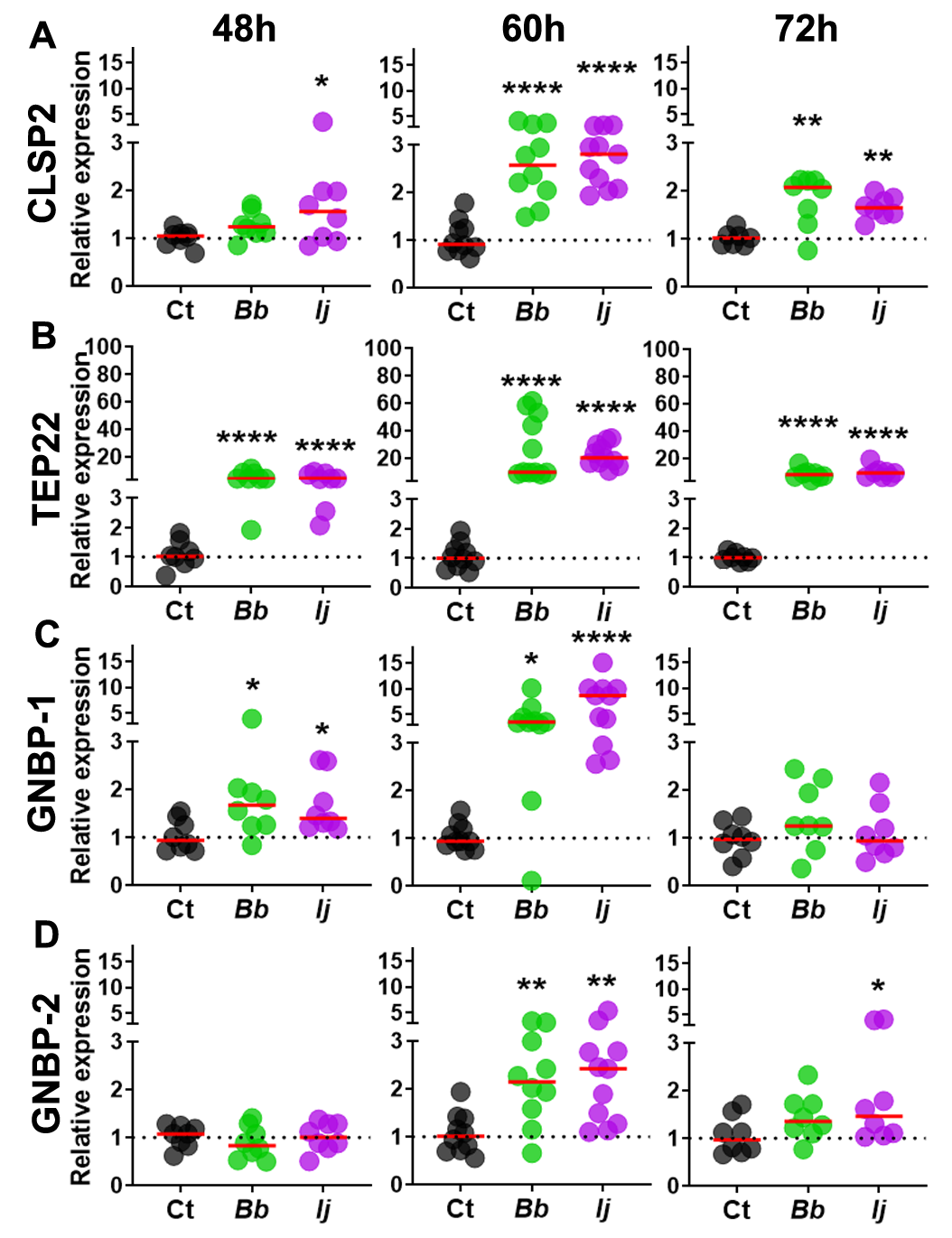

Supplement: Figure S1 — Gene expression profiles in mosquito whole-bodies infected with either B. bassiana or I. javanica, evaluated at 48, 60, and 72 h PI. Induction of (A) CLSP2, (B) TEP22, (C) GNBP-1, and (D) GNBP-2 in Ae. aegypti mosquitoes at the early stages of fungal infection. Each dot represents a pool of five mosquitoes and the horizontal red bar indicates the median level of expression from two independent experiments. The statistical significance of fold change values was determined on log2-transformed values via ANOVA followed by Dunn's multiple comparison test. *P < 0.05, **P < 0.01, ****P < 0.0001. [file Image_1.TIF]

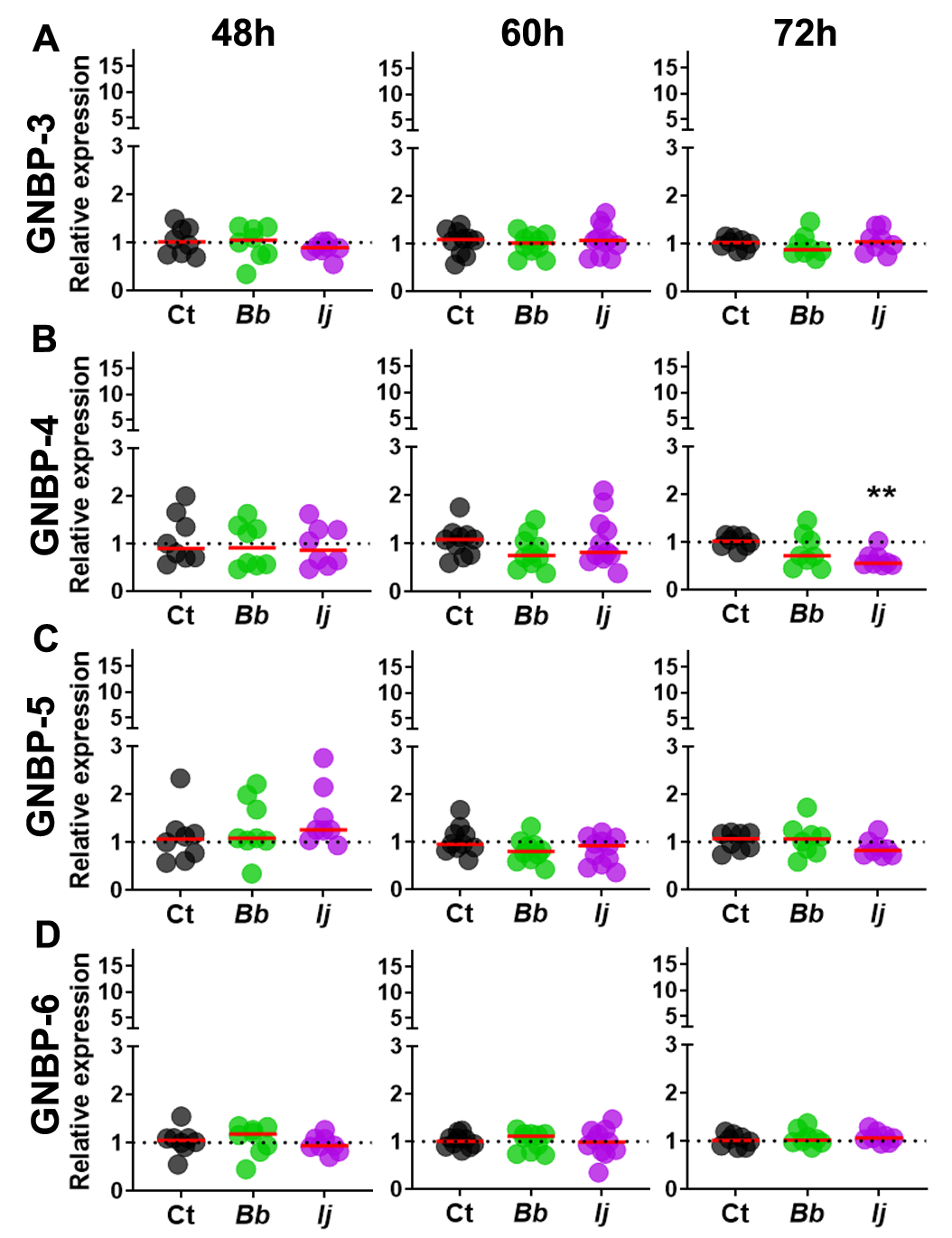

Supplement: Figure S2 — Gene expression profiles in mosquito whole-bodies infected with either B. bassiana or I. javanica, evaluated at 48, 60, and 72 h PI. Induction of (A) GNBP-3, (B) GNBP-4, (C) GNBP-5, and (D) GNBP-6 in Ae. aegypti mosquitoes at the early stages of fungal infection. Each dot represents a pool of five mosquitoes and the horizontal red bar indicates the median level of expression from two independent experiments. The statistical significance of fold change values was determined on log2-transformed values via ANOVA followed by Dunn's multiple comparison test. **P < 0.01. [file Image_2.TIF]

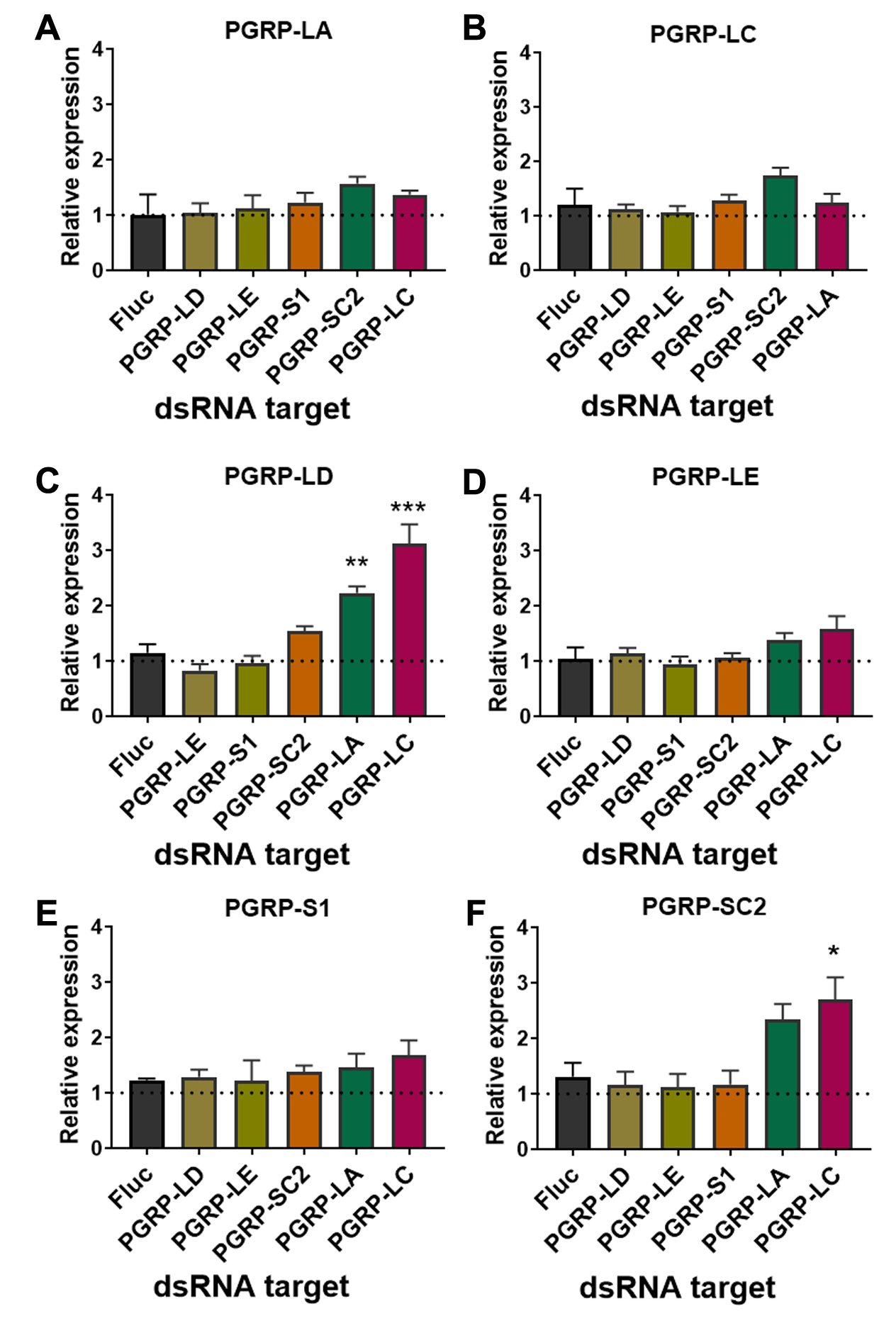

Supplement: Figure S3 — PGRP induction in mosquitoes with single knockdowns of Fluc (control), (A) PGRP-LA, (B) PGRP-LC, (C) PGRP-LD, (D) PGRP-LE, (E) PGRP-S1, or (F) PGRP-SC2. Gene expression profiles were conducted in mosquito whole-bodies at 3-days post dsRNA injection. The statistical significance of fold change values was determined on log2-transformed values via ANOVA followed by Dunn's multiple comparison test. *P < 0.05, **P < 0.01, ***P < 0.001. [file Image_3.TIF]
